# Practical Program Evaluation: Assessing and Improving Planning, Implementation, and Effectiveness

**Published:** 2005-12-15

**Authors:** S. René Lavinghouze

**Affiliations:** Division of Oral Health, Centers for Disease Control and Prevention, Atlanta, Ga

**Figure:**
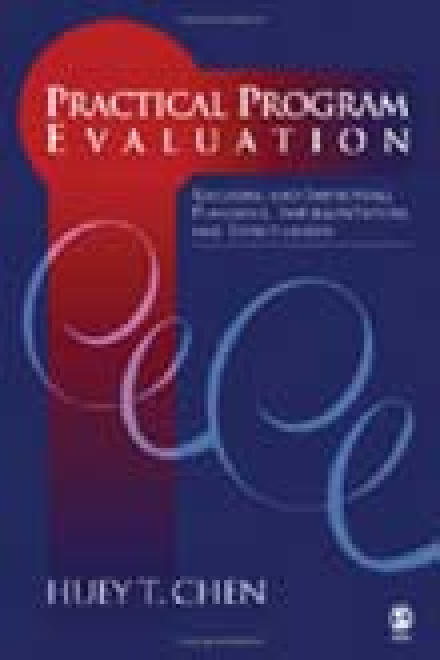


*Practical Program Evaluation: Assessing and Improving Planning, Implementation, and Effectiveness* is an excellent first step toward a much-needed program evaluation taxonomy — one that is particularly useful for those new to evaluation, as well as for seasoned evaluators who would like to encourage understanding of the evaluation process. In this four-part, 11-chapter book, Chen discusses the evaluation process from program planning to outcome assessment. He advocates a theory-driven evaluation approach that supports his taxonomy and provides a thorough review of the theory-driven approach. Chen explains that many evaluation concepts are too vague or ambiguous to apply effectively to actual evaluations. He proposes an evaluation taxonomy to exemplify a holistic approach to evaluation practice. The new taxonomy supports evaluators in their attempts to understand and apply evaluation designs, data collection techniques, and use of evaluation information at a practical program level. This is no small achievement and should be an impetus for additional work in the area of evaluation taxonomy development. The taxonomy and overview of the process are excellent tools for strengthening communication between stakeholders and evaluators. The work is replete with diagrams, examples, and definitions and would be a welcome addition to any evaluation course curriculum.

Chen states that the intended audience for this book is students who have completed an entry-level evaluation course, as well as seasoned evaluators who would like to expand their knowledge and strengthen their practical skills. He acknowledges that many of the terms and definitions presented are not consistent with terms readily found in the current literature, but he encourages the readers to broaden their understanding of evaluation. For example, Chen discards the logic model in favor of his action-model and change-model approach. Although this could be an interesting challenge for seasoned evaluators, it might prove more of a burden for novice evaluators. In the final chapters, Chen also briefly addresses some criticisms of theory-driven evaluation and challenges evaluation practitioners to think about the politics and contributions of evaluation activities.

Chen also skillfully demonstrates that stakeholder theory is a legitimate basis for theory-driven programs and needs to be explained and vetted like established theories of behavior change such as the Health Belief Model. He reminds evaluation practitioners that program theory and the program itself belong to the stakeholders. Chen proposes that the role evaluation practitioners should play varies from one of objective observer to one of a clear partner in the development and design of the program. With these varying roles, the evaluation approach will also change.

As with all the strategies and approaches he addresses, Chen provides a thorough discussion of the positives and negatives of efficiency and effectiveness evaluation. Although the book provides an insightful discussion of evaluation in the program planning, development, and maturation stages, Chen does not discuss evaluation use or discern intended users (program stakeholders). The taxonomy presented can guide evaluation practitioners through the conceptualization and implementation of approaches and methods suited to each stage of a program's development. However, it does not provide adequate guidance for obtaining stakeholder questions and evaluation priorities. Chen does argue for the inclusion of stakeholders throughout the evaluation process and proposes that use of the taxonomy will facilitate discussions between evaluators and stakeholders, but this will be derived from the program theory and stage of development rather than from the intended users and projected uses of the evaluation. Chen's discussion of qualitative, quantitative, and mixed methods still helps students conceptualize potential problems and learn how to address them as they read about the abundance of evaluation approaches available. 

The book is a welcome addition to the expanding literature on evaluation, because it provides an overall conceptualization of the evaluation process from program design to implementation. Although the book is somewhat limited by its lack of discussion about how evaluation results are used, this breakthrough in conceptualization will surely encourage more work in the area.

